# Creating 3D visualizations of MRI data: A brief guide

**DOI:** 10.12688/f1000research.6838.1

**Published:** 2015-08-04

**Authors:** Christopher R. Madan

**Affiliations:** 1 Department of Psychology, Boston College, Chestnut Hill, MA, USA

**Keywords:** MRI visualization, brain mapping, fMRI, image processing, neuroanatomy, ROI

## Abstract

While magnetic resonance imaging (MRI) data is itself 3D, it is often difficult to adequately present the results papers and slides in 3D. As a result, findings of MRI studies are often presented in 2D instead. A solution is to create figures that include perspective and can convey 3D information; such figures can sometimes be produced by standard functional magnetic resonance imaging (fMRI) analysis packages and related specialty programs. However, many options cannot provide functionality such as visualizing activation clusters that are both cortical and subcortical (i.e., a 3D glass brain), the production of several statistical maps with an identical perspective in the 3D rendering, or animated renderings. Here I detail an approach for creating 3D visualizations of MRI data that satisfies all of these criteria. Though a 3D ‘glass brain’ rendering can sometimes be difficult to interpret, they are useful in showing a more overall representation of the results, whereas the traditional slices show a more local view. Combined, presenting both 2D and 3D representations of MR images can provide a more comprehensive view of the study’s findings.

## Introduction

**Figure 1.**
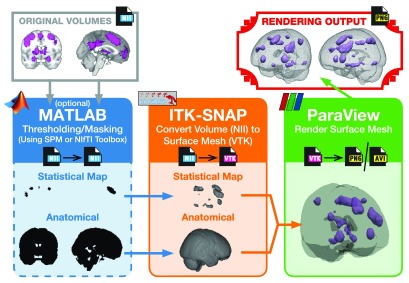
Overview of the processing pipeline.

When presenting and publishing findings of magnetic resonance imaging (MRI) studies, sometimes it is difficult to adequately present the results because they are 3D, while papers and slides can inherently only be 2D. A solution is to create figures that include perspective and can convey 3D information, and the creation of such figures can be readily produced using standard functional magnetic resonance imaging (fMRI) analysis packages, such as SPM ( http://www.fil.ion.ucl.ac.uk/spm/), AFNI ( http://afni.nimh.nih.gov/afni/; with SUMA), and FreeSurfer ( https://surfer.nmr.mgh.harvard.edu), as well as some more specialty programs, such as MRIcroGL ( http://www.mccauslandcenter.sc.edu/mricrogl/), 3DSlicer ( http://www.slicer.org), and Mango ( http://ric.uthscsa.edu/mango/). While these numerous options can provide 3D renderings of MRI data, many of them are unable to provide useful functionality such as visualizing activation clusters that are both cortical and subcortical, i.e., a 3D glass brain. This difficulty increases further if one wants to produce 3D renderings of several activation maps with an identical perspective (e.g., camera angle) or animated renderings (e.g., a rotating 3D glass brain). Here I briefly detail a straightforward approach for creating 3D visualizations of MRI data that work in these scenarios, as well as readily generalize to most other instances. An illustration of this processing workflow is shown in Figure 1. An additional example of making a 3D rendering of traced regions of interest (ROIs) is also outlined.

**Figure 2.**
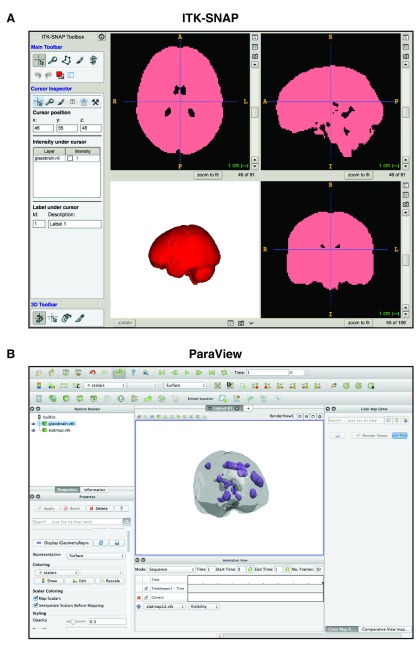
Screenshots of ( **a**) ITK-SNAP and ( **b**) ParaView.

The guide will primarily utilize two programs, ITK-SNAP (v. 3.0.0; Figure 2A; http://www.itksnap.org; Yushkevich *et al.*, 2006) and ParaView (v. 4.3.1; Figure 2B; http://www.paraview.org; Ayachit, 2015). Both programs are available for both Windows and Mac operating systems and are freely available. Data files produced in the examples are provided in the Supplementary material (see Appendix A).

## Methods

### Procedure #1: Visualizing cluster maps in a glass brain

As a first exercise in visualizing MRI data in 3D, we will start with a statistical map. Depending on where your maps are coming from, you may need to apply a height threshold (i.e., *t*- or *Z*-critical) and/or a minimum cluster extend threshold ( *k*). As a starting point and to make this guide more general and more reproducible, I will start with a statistical map obtained from NeuroSynth ( http://www.neurosynth.org; Yarkoni *et al.*, 2011), which will be in NIfTI format (Neuroimaging Informatics Technology Initiative; http://nifti.nimh.nih.gov). Briefly, NeuroSynth conducts automated meta-analyses across thousands of fMRI studies by calculating a frequency metric for how often specific terms are mentioned in the paper (e.g., “memory”, “emotion”) in relation to voxels reported in the results tables. See Yarkoni *et al.* (2011) for further details. As an example of how to obtain thresholded statistical maps from SPM analyses, see Appendix B.

**Figure 3.**
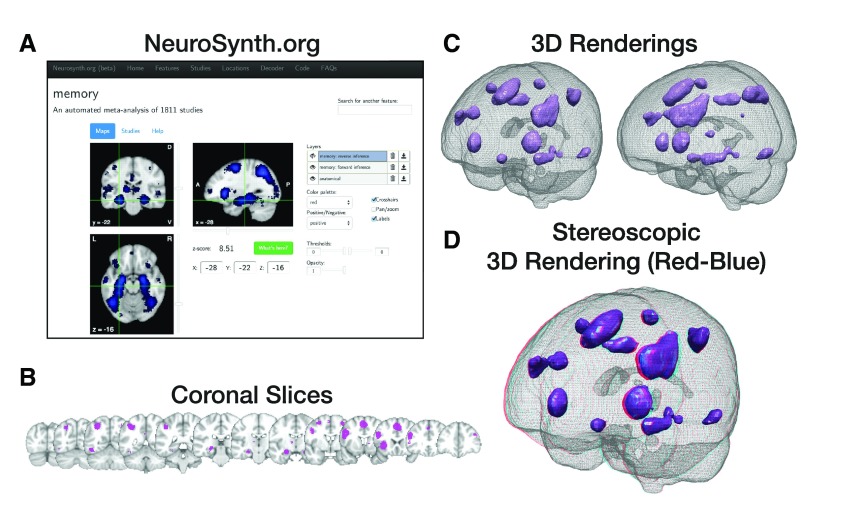
Images of the statistical map used in Procedure #1. ( **a**) Obtaining the statistical map from NeuroSynth.org. ( **b**) Coronal slices of the thresholded activation clusters. ( **c**) 3D renderings of the clusters from two different perspectives. ( **d**) Stereoscopic anaglyph 3D rendering of the first perspective shown in panel C, to be viewed using red-blue 3D glasses.

 For this example I searched the online version of NeuroSynth for the term “memory” ( http://www.neurosynth.org/analyses/terms/memory/; see Figure 3A). I used the “forward inference” map as the example statistical map, along with the anatomical volume provided (obtained by clicking the download buttons displayed to the right of the layer names). For these examples, un-gzip the NIfTI volumes from NeuroSynth. Rename the forward inference map file from memory_pAgF_z_FDR_0̷.0̷1.nii to statmap.nii.

***Obtaining the anatomical ‘glass brain’ image.*** Since the anatomical 3D surface meshes are generally usable, in addition to outlining the steps for creating this glass brain volume, the resulting surface mesh file is also provided as Supplementary material (see Appendix A). While there is an abundance of anatomical volumes in normalized template space, here we will use the one provided on NeuroSynth (click “anatomical” where shown in Figure 2A).

***Obtaining the thresholded cluster image.*** Before the map can be rendered in 3D, both the height and cluster-extent thresholds should be applied. In some fMRI analysis packages this can be output directly (e.g., see Appendix B). If this is the case, export the threshold cluster image and skip to section 1.3; if this is not the case, we will manually apply these thresholds ourselves. Here I will use examples of how to manually apply these thresholds using MATLAB (R2013a; The MathWorks Inc., Natick, MA) and SPM8 ( http://www.fil.ion.ucl.ac.uk/spm/; Wellcome Department of Cognitive Neurology, UCL, London, UK), though other packages are able to do this as well. (See Madan, 2014, for an introductory guide to MATLAB.)

Manually applying the height and cluster-extent threshold is a bit cumbersome. Using the imcalc function in SPM, we can easily apply a height threshold to our volume by outputting a binary volume, where the voxel intensity statistic (i.e., from a *t*-, *F*-, or *Z*-statistic map) is above the threshold. Since our NeuroSynth memory image has a large number of highly significant clusters, we will threshold our statmap.nii to isolate the voxels where the statistic ( *Z*-value) is above 12 (MATLAB code shown below). In the current case, lower thresholds yielded large clusters, which made the figure less interpretable (i.e., many regions comprised a single cluster, making it difficult to view the topology of the regions from the 3D view). When plotting results from your own fMRI study, you would likely use a threshold around 3 for the *t*- or *Z*-statistical map (corresponding to approximately *p*<.001).



                        >> spm_imcalc_ui(
                        'statmap.nii',
                        'statmapH.nii',
                        'i1>12'
                        );
                    


To apply the cluster-extent threshold, we will use the nii_threshreslicecluster function (freely available from http://www.mccauslandcenter.sc.edu/CRNL/tools/spm8-scripts) to isolate clusters of voxels of at least a minimum volume of 400 mm ^3^. Again, this value can be adjusted, and usually would be set higher than you would use for your statistical analyses, as the 3D rendering is intended more to provide a global view of the significant clusters, and is encumbered by the inclusion of many small clusters. A volume of 400 mm ^3^ corresponds to 50 voxels where the voxel size is 2 mm-isotropic. The function can also apply height thresholds, but it thresholds rather than binarizes the image (i.e., converting it to a mask), which is not as useful for our current purposes.



                        >> nii_threshreslicecluster (
                        'statmapH.nii'
                        ,
                        'statmapH.nii'
                        ,
                        .5
                        ,
                        40̷0̷
                        );
                    


The output file from this command will automatically be named rstatmapH.nii, rename it to statmapThresh.nii.

The height threshold should also be applied to the anatomical volume, anatomical.nii, but the cluster threshold is unnecessary. The resulting output file will be named anatomicalH.nii, rename it to glassbrain.nii.

***Convert to VTK.*** To visualize the NIfTI volumes in 3D, we need to convert the voxel data into a 3D surface mesh in the VTK (Visualization ToolKit) format. Designed for anatomical tracing, ITK-SNAP includes this functionality. The simplest way to do so is to load each volume as *both* the main volume and as the segmentation volume. If you use the structural volume as the main volume and the statistical map as the segmentation, you may have issues with the bounding boxes not matching. Since we will move to another program with our 3D surfaces, it does not matter if the bounding boxes match or not.

Make sure that the volume is loaded correctly, as shown in Figure 2A. While ITK-SNAP can render 3D volumes, as shown in the bottom left portion of the screenshot shown in Figure 2A, its rendering options are limited. For instance, if you want to render several volumes in 3D from a consistent perspective/camera angle, ITK-SNAP is unable to accommodate; while ITK-SNAP *can* temporarily store camera information, this perspective information is lost if the program is closed or crashes, and it cannot be saved for later use nor can it be manually specified. As a result, it will be impossible to obtain the *exact* same camera angle. To rectify this shortcoming, we will make our 3D renderings in ParaView, which also has additional useful features. To export the meshes in VTK format from ITK-SNAP, use the menus to navigate to Segmentation, then “Export as Surface Mesh…”. Next, choose “Export meshes for all labels as a single scene” and save the file as a “VTK PolyData File”. In the current example there is only one surface mesh in each volume, but this is not always the case, such as in the ROI example discussed later. Note, it is possible to export volume data, rather than surface mesh data, as a VTK file in ITK-SNAP, but these files will not work with ParaView in the next step. If your VTK file does not work, double check that it was correctly exported as a surface mesh.

Repeat these steps for both the statistical map and anatomical volume.

***Render in 3D.*** Start ParaView and open your two new VTK files within the same scene. ParaView can be a bit overwhelming at first, but it has many useful features for rendering and setting up the camera. With some adjustment of the colors and opacity for the two surfaces, it should be fairly easy to produce a set up in ParaView similar to Figure 2B. You can rotate the camera manually using the mouse, and can reset the camera position with the buttons labelled “+X” through “−Z”. When the scene state is saved, the camera position is preserved in the scene file, allowing you to easily load another statistical map at a later time. The scene as a whole can be saved by selecting “File” then “Save State…” (PVSM format). The final renderings produced here are shown in Figure 3C, corresponding to the series of coronal slices shown in Figure 3B. Renderings can be saved using either “File” then “Save Screenshot…” or “Export Scene…”. Screenshots will always be exported as raster (i.e., pixel) images, while ‘exported scenes’ are vector/polygon based. Note that exported PDFs can also be based on “rasterize 3D geometry” (there is a checkbox). If you are unsure what you require, a screenshot is likely sufficient, but do try and experiment to find out what settings best meet your needs, as this overview of ParaView’s functionality is far from comprehensive.

ParaView can also render stereoscopic 3D figures (e.g., anaglyph [red-blue], side-by-side) with a variety of 3D-compatible glasses options. An example of a red-blue stereoscopic render is shown in Figure 3D.

### Procedure #2: Visualizing anatomical ROIs

**Figure 4.**
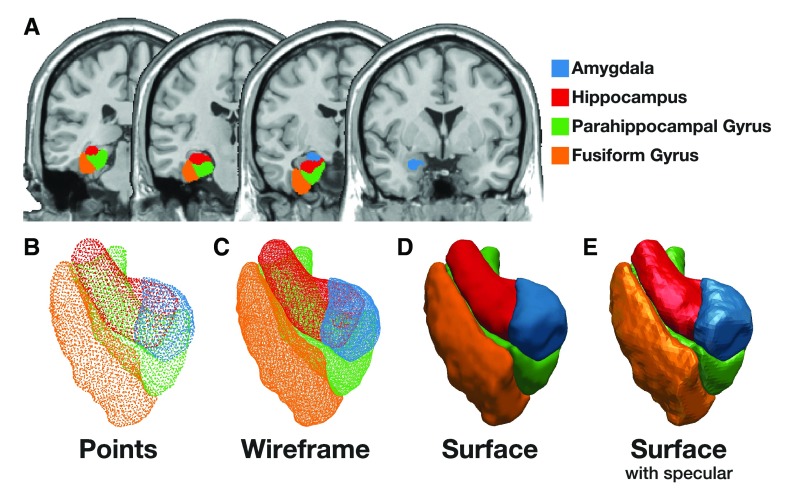
Images of the anatomical ROIs used in Procedure #2. ( **a**) Coronal slices of the anatomical ROIs. Panels B-E depict different 3D rendering settings of the ROIs, ( **b**) points along the surface of the ROIs, ( **c**) wireframe, ( **d**) surface, and ( **e**) surface rendering with specular. Note that the structural image used in panel A is from a different source than the traced ROIs, and thus they do not perfectly align.

***Obtain ROI volume.*** For this example, I extracted several regions of the medial temporal cortex (hippocampus, amygdala, parahippocampal gyrus, fusiform gyrus) from the right hemisphere of the Hammers *et al.* (2003) maximum probability atlas (n30r83; http://biomedic.doc.ic.ac.uk/brain-development/index.php?n=Main.AdultMaxProb). Regions were extracted using the imcalc tool included in SPM8, such that each ROI corresponded to a unique intensity value (1=hippocampus, 2=amygdala, 3=parahippocampal gyrus, 4=fusiform gyrus):



                        >> spm_imcalc_ui({
                        'Hammers_mith_atlas_n30r83_SPM5.nii'
                        },
                        'roiMTL.nii'
                        ,
                        '(i1==1)*1 + (i1==3)*2 + (i1==9)*3 + (i1==15)*4'
                        );
                    


The ROI is shown plotted over a structural volume in Figure 4A.

***Convert to VTK.*** As before, use ITK-SNAP to load the NIfTI volume and convert it to a VTK surface mesh. If you have multiple surfaces in the same volume, as we do here, be sure to select “Export meshes for all labels as a single scene” when exporting the surfaces.

***Render in 3D.*** Start ParaView and load the VTK file, as done previously. As shown in Figure 4B–E, the volumes can be rendered as points, wireframes, and surfaces. Furthermore, many settings can be customized to adjust the rendering properties, such as the lighting/reflectance properties shown in Figure 4D and 4E.

ParaView can also create cameras that move over time, allowing for the generation of animations of the structures rotating. This can be done using the “Animation View” panel in the bottom-center of ParaView: select “Camera”, “Orbit”, and then “+”. The default settings for the camera positions are usually sufficient. If desired, the camera path can also be edited afterwards by inputting specific coordinates (the best way to preview the path is to simply press ‘play’ at the top and see how it looks). Even without rendering the animation itself, having a camera path allows for later reproduction of 3D renderings from the same camera positions.

Using a camera path, an animation can be rendered by going “File”, “Save Animation”. An example rendered video is shown in Movie 1. (Note, videos here were re-compressed with Handbrake [ https://www.handbrake.fr; freely available for Windows and Mac] to reduce their file size).

Movie 1. Rotating 3D animation of the anatomical ROI with the same render settings as used in Figure 4E. Movie 1. Rotating 3D animation of the anatomical ROI with the same render settings as used in Figure 4E. Click here for additional data file. Copyright: © 2015 Madan CR 2015 Data associated with the article are available under the terms of the Creative Commons Zero "No rights reserved" data waiver (CC0 1.0 Public domain dedication).

## Additional examples

**Figure 5.**
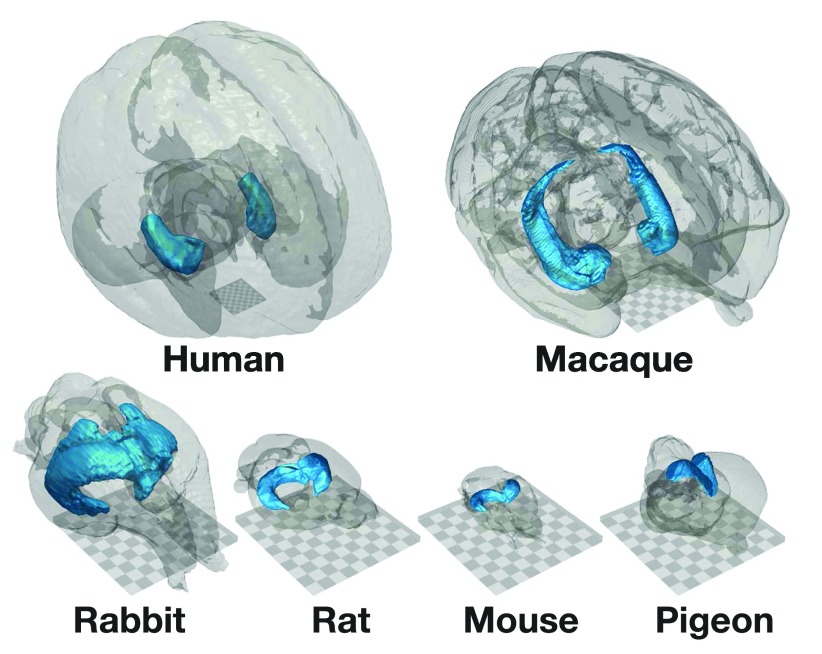
Comparative neuroanatomy of whole-brain and hippocampal brain volumes. The square grid included in each panel measures 20 mm across, with each grid square subtending 2 mm. See Appendix C for details and references describing each brain atlas.

Using the techniques discussed thus far, it is possible to create an image such as that shown in Figure 5, where the hippocampus is shown within a glass brain for a number of different species, using freely available brain atlases. Each panel was rendered separately, but all of the surface meshes were loaded into the same scene in ParaView. By additionally adding a plane with a checkerboard texture, it is also easy to present the scale of the structures. See Appendix C for details regarding each of the brain atlases.

**Figure 6.**
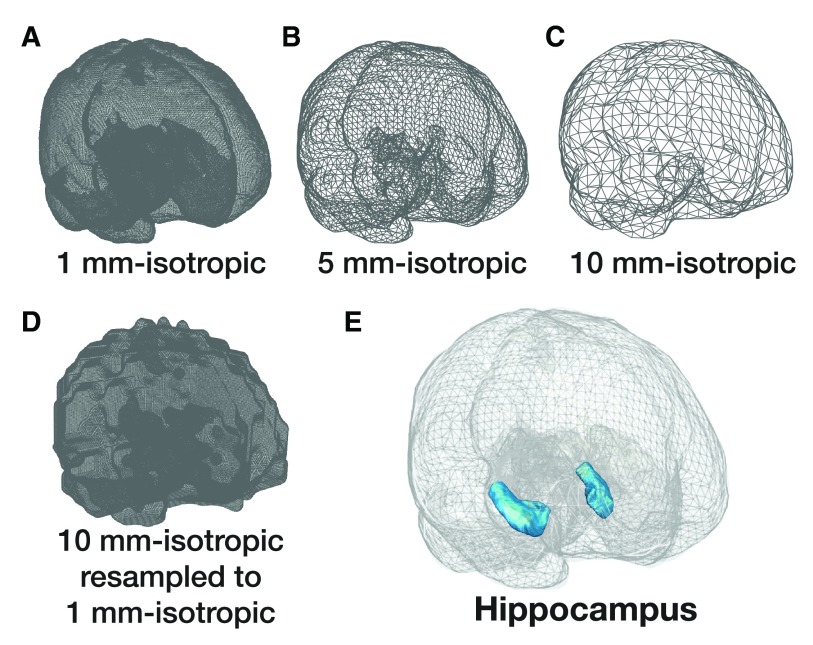
Wireframes of brain images of different resolutions. ( **a**) Wireframe rendering of brain surface mesh produced from the Hammers *et al.* (2003) atlas, which originally has voxel size of 1 mm-isotropic. Panels **B**- **C** show the wireframes of surfaces meshes made after first downsampling the volume to 5 or 10 mm-isotropic, respectively, resulting in less dense wireframes. ( **d**) For demonstration purposes, the result of upsampling the 10 mm-isotropic mesh (panel C) back to 1mm-isotropic. ( **e**) Rendering produced by combining the surface meshes used in panels A-C, along with an anatomical ROI of the hippocampus.

 With a few additional steps, more intricate 3D renderings can also be produced. For instance, if the anatomical volume is down-sampled while in NIfTI format, the resulting surface mesh is less dense and can be rendered as a wireframe, as shown in Figure 6A–C. For demonstration purposes, if the lower-resolution anatomical volume was subsequently up-sampled, the resulting high-density mesh is ‘blocky’ ( Figure 6D). If a combination of different densities of anatomical surface meshes are used together, e.g., the meshes from Figure 6A–C, along with a ROI, a rendering such as Figure 6E can be produced.

**Figure 7.**
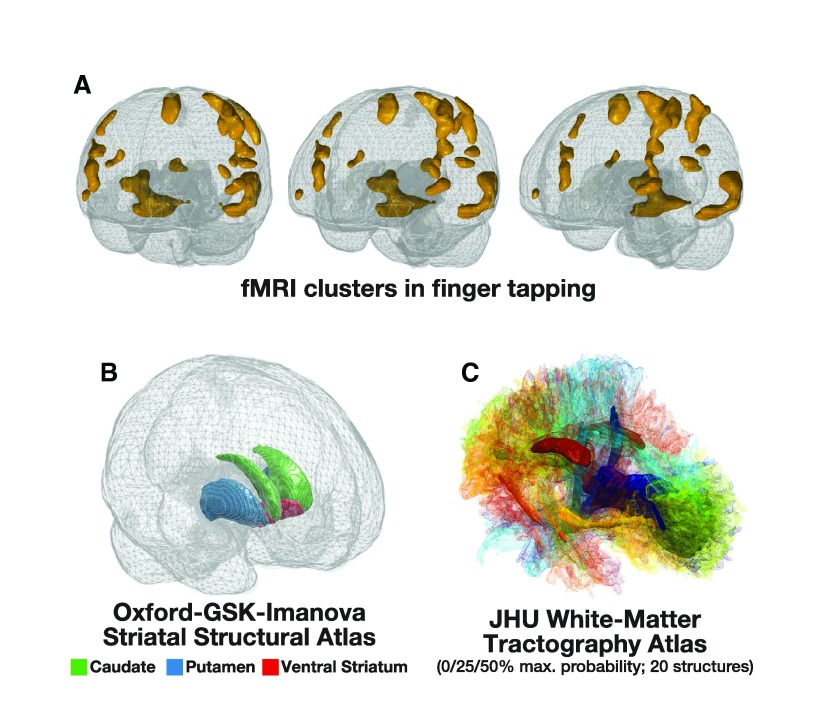
Additional examples of 3D renderings of MRI data. ( **a**) Glass brain rendering of fMRI clusters associated with finger tapping, based on data from Gorgolewski *et al.* (2013), from three perspectives. ( **b**) Glass brain rendering of the anatomical ROIs included in the Oxford-GSK-Imanova Structural Striatal Atlas ( **c**) 3D rendering of the structures included in the JHU white-matter tractography atlas, with different mesh properties used for the 0%, 25%, and 50% probability estimates from the maximum probability volumes. See main text for additional details on the sources of the MRI data.

Figure 7 and Movie 2 show a few additional rendering examples from freely available data. fMRI activity related to finger tapping is shown in Figure 7A, with data obtained from Gorgolewski *et al.* (2013; http://www.neurovault.org/collections/63/; full dataset available at: http://www.openfmri.org/dataset/ds000114). Striatal anatomy is shown in Figure 7B (Oxford-GSK-Imanova Structural Striatal Atlas, from FSL; Tziortzi *et al.*, 2011; http://fsl.fmrib.ox.ac.uk/fsl/fslwiki/Atlases/striatumstruc). DTI tractography showing 20 structures at varying levels of probability estimates is shown in Figure 7C and Movie 2 (JHU white-matter tractography atlas, from FSL; Hua *et al.*, 2008).

Movie 2. Rotating 3D animation of the white-matter tractography atlas with the same render settings as used in Figure 8C. Movie 2. Rotating 3D animation of the white-matter tractography atlas with the same render settings as used in Figure 8C. Click here for additional data file. Copyright: © 2015 Madan CR 2015 Data associated with the article are available under the terms of the Creative Commons Zero "No rights reserved" data waiver (CC0 1.0 Public domain dedication).

## Conclusion

Though a 3D ‘glass brain’ rendering of fMRI activations can sometimes be difficult to interpret, they are useful in showing a more overall representation of which regions are activated, whereas the traditional slices show a more local view of the results. When the goal is to show anatomical structures, 3D figures are definitively more useful in conveying the 3D structure of the regions, as shown in the examples here. Combined, 2D and 3D representations of MR images can provide a more comprehensive view of the results, particularly when at least two 3D perspectives are shown, allowing for some ability to provide depth information.

## Data availability

The data referenced by this article are under copyright with the following copyright statement: Copyright: © 2015 Madan CR

Data associated with the article are available under the terms of the Creative Commons Zero "No rights reserved" data waiver (CC0 1.0 Public domain dedication). 

*Figshare:* Movie 1. Rotating 3D animation of the anatomical ROI with the same render settings as used in Figure 4E. doi: 10.6084/m9.figshare.1499152 ( Madan, 2015a).

*Figshare:* Movie 2. Rotating 3D animation of the white-matter tractography atlas with the same render settings as used in Figure 8C. doi: 10.6084/m9.figshare.1499153 ( Madan, 2015b).

## Supplementary material

**Data files for ‘Creating 3D visualizations of MRI data: A brief guide’.** The supplementary files are divided into two folders, “neurosynth” and “hammers”; see Appendix A.

## Appendix A: Notes on supplemental data files

The supplementary files are divided into two folders, “neurosynth” and “hammers”, corresponding to the first and second procedures described here, respectively.

neurosynth/memory_pAgF_z_FDR_0̷.0̷1.nii.gz

Original statistical map used in Procedure #1, as obtained from NeuroSynth. Shown in Figure 3A.

neurosynth/statmapThresh.nii

Statistical map after both height and cluster-extent thresholds had been applied. Shown in Figure 3B.

neurosynth/statmapThresh.vtk

Statistical map converted to a surface mesh. Used in Figure 3C–D.

neurosynth/glassbrain.vtk

Anatomical image obtained from NeuroSynth, after being thresholded and converted to a surface mesh. Used in Figure 3C–D.

hammers/brainmask.vtk

Anatomical image obtained from Hammers *et al.* (2003), after being thresholded and converted to a surface mesh. Used in Figure 6 and Figure 7A.

hammers/brainmask_5mm.vtk

Similar surface mesh as brainmask.vtk, but first re-sampled to 5 mm-isotropic voxels prior to conversion to a surface mesh. Shown in Figure 7B.

hammers/brainmask_10̷mm.vtk

Similar surface mesh as brainmask.vtk, but first re-sampled to 10 mm-isotropic voxels prior to conversion to a surface mesh. Shown in Figure 7C.

## Appendix B: Obtaining threshold statistical maps in SPM

In SPM, this would correspond to an spmT_XXXX.img or spmF_XXXX.img file. The simplest approach to thresholding this image is to have SPM output the threshold image, which was introduced in builds of SPM8. To do so, after loading the results for the desired statistical contrast, in the left-bottom panel, select “save” then “all clusters (binary)”. This will output an image that is a mask corresponding to all of the suprathreshold voxels, after both the height and cluster-extent thresholds have been applied.

Alternatively, the height and cluster-extent thresholds can be manually applied to an image, as discussed in Procedure #1. The *T*-value height threshold, corresponding to the desired *p*-value threshold, is reported in the results window when viewing the clusters. This height threshold can easily be applied using the imcalc tool, as described in section 1.2. Subsequently, the cluster-extent threshold would also need to be applied.

## Appendix C: Details on comparative neuroanatomy figure

The comparative neuroanatomy figure shown in Figure 6 was created using atlases from a number of species, each from a different atlas. Three of the atlases were obtained from the Scalable Brain Atlas ( http://scalablebrainatlas.incf.org; Bakker *et al.*, 2015; Majka *et al.*, 2012): macaque, rat, and mouse.

The human atlas used was developed by Hammers *et al.* (2003) and is in MNI/ICBM 152 space, based off maximum probabilities over twenty individuals. The macaque atlas was developed by Rohlfing *et al.* (2012), where the INIA19 template was created from T1 images along with labelled maps from the NeuroMaps atlas. The rabbit atlas was developed by Muñoz-Moreno *et al.* (2013) based off the New-Zealand rabbit, from maximum probabilities across ten individuals. The rat atlas was developed by Calabrese *et al.* (2013) using Wistar rats and incorporated eight MR protocols as well as multiple scanning sessions, in order to quantify neuronal changes as a function of normal development. The mouse atlas was developed by Johnson *et al.* (2010), where the Waxholm Space was created from C57BL/6 mice and relied on a combination of three MR protocols and conventional Nissl histology. The pigeon atlas was developed by Güntürkün *et al.* (2013) using MR and CT images along with histology.

For all atlases, the whole-brain mask was extracted as the glass brain using the third-party NIfTI Toolbox for MATLAB ( http://www.mathworks.com/matlabcentral/fileexchange/8797-tools-for-nifti-and-analyze-image), as well as the ROI(s) corresponding to the hippocampus. When necessary, minor pre-processing was applied, such as re-slicing to down-sample the volume’s resolution and mirroring of unilateral atlases to create a bilateral volume.
