# Supplementary material for: Creating 3D visualizations of MRI data: A brief guide
Source: F1000Res. 2015 Aug 4;4:466. [Version 1] doi: 10.12688/f1000research.6838.1 (PMC4648228; doi:10.12688/f1000research.6838.1)
Supplement: Peer review history

# Peer review: Referee response for version 1

**DOI:** 10.5256/f1000research.7352.r9792

**Published:** 2015-09-07

**Authors:** Matthew Wall

**Affiliations:** 1 Division of Brain Sciences, Imperial College London, London, UK

This is a very useful guide to an important issue that is currently largely overlooked in the literature; producing high-quality presentations of brain imaging results that are informative, clear, and useful. The article is comprehensive and easy to follow, and the examples provided are appropriate, and produce very attractive images. This is an extremely useful paper that deserves wide readership in the field.

While I agree with the author that ‘glass-brain’ visualisations are extremely useful for providing a comprehensive overview of patterns of brain activity in fMRI experiments, that doesn’t mean that conventional 2D slice views are not also useful. In fact, 2D views of particular activation clusters are really the only way to get a good idea of the precise position of a cluster, in relation to the sulcal/gyral anatomy, which is often important. An optimal strategy for comprehensive visualisation and localisation might then be to combine 2D and 3D views of results in the same figure. The author has done this more-or-less in Figure 3 (which includes coronal slices), but I wonder if perhaps an additional example figure which combines 2D and 3D views might be helpful? Perhaps as an example of the kinds of ‘real’ figures that could be produced for publications and presentations.

Minor points of grammar, etc.:

Abstract:

"they are useful in showing a more overall representation of the results" More overall? Somewhat clumsy; replace with "more general" or just "overall".

Page 2 first paragraph: "Here I briefly detail a straight- forward approach for creating 3D visualizations of MRI data that work in these scenarios, as well as readily generalize to most other instances." Something wrong with the tenses here; would suggest: "Here I briefly detail a straight- forward approach for creating 3D visualizations of MRI data that works in these scenarios, and also readily generalizes to most other instances."

Page 4. Section on obtaining and thresholding the images. Fine, but the procedure outlined here is pretty cumbersome, as the author admits! This procedure might be optimal for those who use SPM as their primary analysis tool, but the 'fslmaths' function included with FSL could achieve this in a single command-line entry. Maybe include a sentence saying something like "Other options for thresholding are available, such as the basic functions included with FSL."

I have read this submission. I believe that I have an appropriate level of expertise to confirm that it is of an acceptable scientific standard.

# Peer review: Referee response for version 1

**DOI:** 10.5256/f1000research.7352.r9899

**Published:** 2015-08-10

**Authors:** Anders Eklund

**Affiliations:** 1 Department of Biomedical Engineering, Linköping University, Linköping, Sweden

I think that this is a useful paper. Here are some minor comments

You do not mention anything about multiple comparisons for the thresholding. I understand that these visualizations are mainly for obtaining a better understanding of the brain activation, but it would still be nice to mention the problems of multiple testing. For cluster level inference, I prefer if a cluster p-value threshold is used, and not an arbitrary cluster size like 400 mm ^3^ or 50 voxels. Cluster p-values can be obtained through parametric methods (Gaussian random field theory, available in SPM and FSL) or non-parametric methods (permutation testing, available in SnPM, FSL and BROCCOLI). I know that a very common approach is to use a cluster defining threshold of p = 0.001 or p = 0.005 (uncorrected for multiple comparisons), combined with an arbitrary cluster size threshold of 10 voxels. Such approaches should in my opinion be avoided, since the method is ad-hoc; it is impossible to know what the (corrected) p-value is for the combined procedure.

The following paper may be of interest:

Choong-Wan Woo, Anjali Krishnan, Tor D. Wager, Cluster-extent based thresholding in fMRI analyses: Pitfalls and recommendations, NeuroImage, Volume 91, 1 May 2014, Pages 412-419, ISSN 1053-8119, http://dx.doi.org/10.1016/j.neuroimage.2013.12.058

-------

You may mention two additional pieces of software, pysurfer and MevisLab.

Pysurfer is a python tool for visualizing cortical surface representations

https://pysurfer.github.io/

MevisLab is a free software that can be used for image processing and visualization. MevisLab includes functions from the libraries VTK and ITK, and it is easy to setup more advanced volume rendering pipelines, where you for example have several volume renderers, clip planes and more advanced transfer functions.

http://www.mevislab.de/

-------

You do not mention anything about visualization research regarding fMRI. A more advanced way to visualize brain activation is to treat the activation as a light source in the anatomical volume, making the activity "glow" from the inside. You could include some of the following papers.

Nguyen, T. K., Eklund, A., Ohlsson, H., Hernell, F., Ljung, P., Forsell, C., Andersson, M., Knutsson, H., Ynnerman, A., Concurrent Volume Visualization of Real-time fMRI, Proceedings of the 8th IEEE/EG International Conference on Volume Graphics, 53-60, 2010, http://dx.doi.org/10.2312/VG/VG10/053-060

Janoos, F., Nouanesengsy, B., Machiraju, R., Shen, H. W., Sammet, S., Knopp, M. and Mórocz, I. Á. (2009), Visual Analysis of Brain Activity from fMRI Data. Computer Graphics Forum, 28: 903–910. doi: 10.1111/j.1467-8659.2009.01458.x

Jainek, W. M., Born, S., Bartz, D., Straßer, W. and Fischer, J. (2008), Illustrative Hybrid Visualization and Exploration of Anatomical and Functional Brain Data. Computer Graphics Forum, 27: 855–862. doi: 10.1111/j.1467-8659.2008.01217.x

Rieder, C., Ritter, F., Raspe, M. and Peitgen, H.-O. (2008), Interactive Visualization of Multimodal Volume Data for Neurosurgical Tumor Treatment. Computer Graphics Forum, 27: 1055–1062. doi: 10.1111/j.1467-8659.2008.01242.x

I have read this submission. I believe that I have an appropriate level of expertise to confirm that it is of an acceptable scientific standard.

# Peer review: Referee response for version 1

**DOI:** 10.5256/f1000research.7352.r9794

**Published:** 2015-08-06

**Authors:** Jens Foell

**Affiliations:** 1 Department of Psychology, Florida State University, Tallahassee, FL, USA

The manuscript in question describes different methods to visualize data acquired through MRI/fMRI scans in a three-dimensional manner. This is something that is sometimes done in current neuroimaging research, but that is rarely done in a standardized manner, which makes this guide timely and relevant. In many cases, researchers choose to use 2D images instead, which can sometimes distort or omit information, as fMRI depictions are derived from an inherently 3-dimensional signal. The current manuscript separately describes ways to visualize clusters of activation (i.e. activation as it would be found when running an fMRI experiment) and anatomical regions of interest. It also provides hyperlinks to download relevant visualization software. The author goes into sufficient detail to include, for example, information on price and OS compatibility of different software packages. Also, the text provides details about how to create the images within a particular software package, or functions that increase user efficiency. Information like this, in addition to several informative illustrations in the manuscript, will make this text particularly useful for many people working in neuroimaging, and I am convinced that the publication of this manuscript will lead to a fruitful online discussion about the best ways to visualize and report 3D brain data.

The title, abstract, and structuring of the manuscript are well-written and appropriate for its purpose as a brief guide.

Overall, this concise and informative guide is useful, interesting, and well-written. I recommend its indexing after some very minor comments (listed below) have been addressed to increase the readability of the manuscript.

Minor suggestions:

1. While the term ‘3D’ could be considered to be a household word, I would still recommend to spell it out as ‘three-dimensional (3D)’ or ‘3-dimensional (3D)’ the first time the term is used in the text.
2. Likewise, the term ‘glass brain’ is intuitive, but not always used in the same way by all researchers. A quick description of the concept at the first mention of the term in the text would make the manuscript more accessible to the general reader.

I have read this submission. I believe that I have an appropriate level of expertise to confirm that it is of an acceptable scientific standard.
